# Utility of Red Cell Distribution Width as a Prognostic Factor in Young Breast Cancer Patients

**DOI:** 10.1097/MD.0000000000003430

**Published:** 2016-04-29

**Authors:** Du-Ping Huang, Rui-Min Ma, You-Qun Xiang

**Affiliations:** From the Department of Oncology, the First Affiliated Hospital of Wenzhou Medical University, Wenzhou, China.

## Abstract

The prognosis of breast cancer occurs in young women is usually poor. Red cell distribution width (RDW), 1 of many routinely examined parameters, has recently been proposed as a prognostic marker in solid tumors. The aim of our study was to assess the predictive value of RDW for survival in young women with breast cancer.

We reviewed 203 consecutive young female patients (under 40) with invasive breast cancer diagnosed at the First Affiliated Hospital of Wenzhou Medical University between January 2008 and December 2012. Preoperational RDW, clinicopathological information, and prognostic data were collected. RDW levels were divided into 2 groups: 161 patients with low RDW (≤13.75%) and 42 patients with high RDW (>13.75%). Clinicopathological differences between the 2 groups were calculated by chi-squared test and Wilcoxon rank-sum test. Kaplan–Meier survival analysis and Cox proportional hazard regression analyses were used to examine the effect of RDW on survival.

We found that high RDW was significantly associated with larger tumor size (*P* = 0.002), positive lymph node metastases (*P* = 0.011), and advanced stages (*P* = 0.004). Patients with high RDW showed significantly lower disease-free survival (DFS; *P* < 0.001) and lower overall survival (OS) rate (*P* < 0.001) than patients with low RDW. Moreover, the Cox regression multivariate analysis revealed that high pretreatment DRW was independently correlated with poor DFS and OS, with hazard ratio 4.819 (95% confidence interval [CI] 2.291–10.138, *P* < 0.001) and 5.887 (95% CI 1.666–20.802, *P* = 0.006), respectively.

In conclusion, our study demonstrated that pretreatment RDW may be associated with DFS and OS in young women with breast cancer. Further validation and feasibility studies are required before the result of our study can be considered for clinical practice.

## INTRODUCTION

Breast cancer diagnosed in women age ≤40 years is a relatively rare disease; however, breast cancer is the leading cause of cancer death in young women.^1,2^ Young women are more likely to develop more aggressive subtypes of breast cancer and to have poorer survival than their older counterparts.^3–5^ The reasons for worse prognosis in young women are complex and are likely related to multiple factors.^6,7^

It is now widely recognized that smouldering inflammation in the tumor microenvironment plays a pivotal role in the initiation, progression, and progression of cancer.^8–10^ Red cell distribution width (RDW) is a measurement of variability and size of erythrocytes, and is performed routinely as part of a complete blood cell count. As an easy-to-measure inflammatory marker of systemic inflammatory response, RDW has been reported in many pathophysiological conditions including cardiovascular disease and generally increased progressive inflammations.^11–16^ Recently, RDW is increasingly being recognized to have an important role in carcinogenesis, tumor progression, and prognosis.^16–21^ Moreover, a previous study indicated that RDW may be a potential biomarker of the activity of breast cancer. However, there has been no report on the prognostic value of RDW in young women with breast cancer. Therefore, the aim of this study was to investigate the association between RDW, disease-free survival (DFS), and overall survival (OS) in young women with breast cancer.

## MATERIALS AND METHODS

### Patients

**FIGURE 1:**
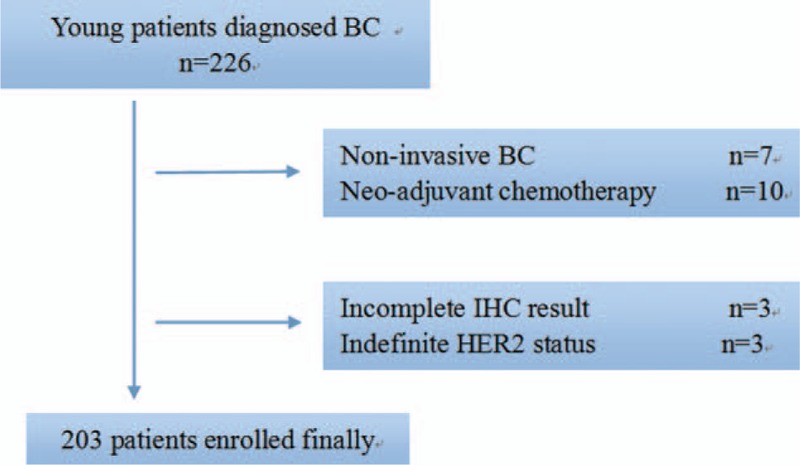
Two hundred twenty-six consecutive young patients were diagnosed and completed the treatment of breast cancer; finally, 203 patients were eligible for analysis.

We reviewed young female patients (age ≤ 40) who were pathologically diagnosed invasive breast cancer (T1–4 N0–3 M0) and treated at the First Affiliated Hospital of Wenzhou Medical University in China, between January 2008 and December 2012. Exclusion criteria as follows: noninvasive breast cancer or stage IV breast cancer or inflammatory breast cancer; taking preoperatively treatment including neo-adjuvant chemotherapy (CT); patients with lack of information on pathologic or laboratory results; and patients with systemic inflammatory or chronic disease such as heart failure, systemic lupus erythematosus, hematological disorders, liver cirrhosis, and coronary artery disease. Hence, 203 consecutive patients were enrolled (Figure 1). Pathologic and laboratory data of all patients were collected from electronic medical records. The research protocol was approved by the Ethics Committee of the First Affiliated Hospital of Wenzhou Medical University, and written informed consent was obtained from every patient. They were followed up till June 2015 to obtain survival information.

### Pathological and Immunohistochemical Criteria

The pathological results were evaluated according to AJCC6 criteria by more than 2 associate chief physicians. Immunohistochemical (IHC) standard referred to St. Gallen version 2013: estrogen receptor (ER) positive defined ≥1%; progesterone receptor (PR) positive meant ≥20%; and human epidermal growth factor receptor-2 (HER-2) over-expression considered as IHC 3+. 2+ of HER-2 were further subjected to fluorescence in situ hybridization assays.

### Laboratory Data

**FIGURE 2:**
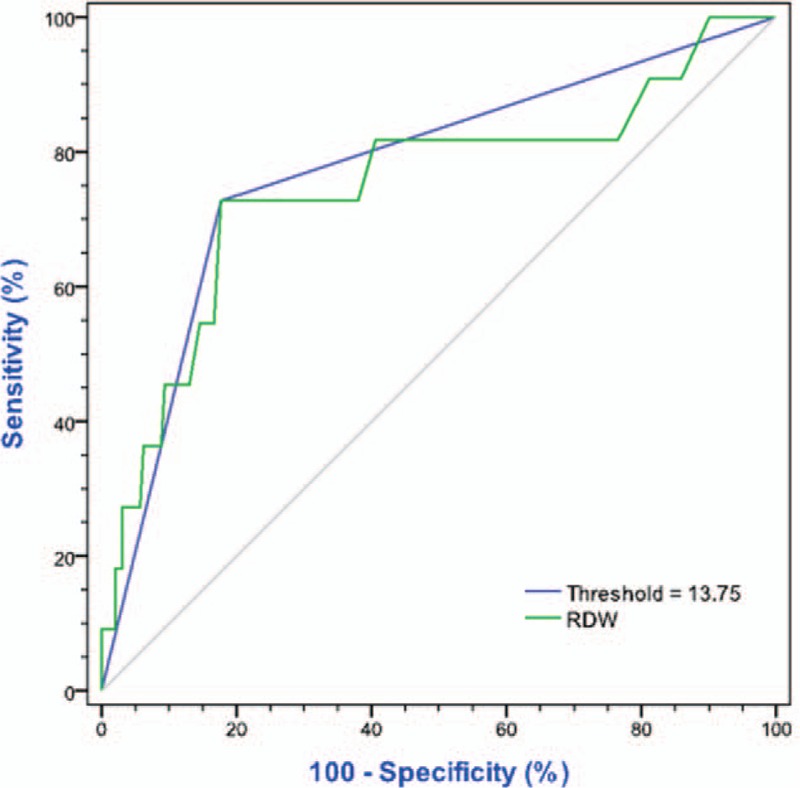
Receiver operating characteristics curve (ROC) analysis based on RDW for OS. In this model, AUC was 0.750 (95% CI 0.573–0.927, *P* = 0.005). When threshold = 13.75%, the AUC = 0.775 (*P* = 0.002), and the sensitivity = 72.7% and specificity = 82.3%. AUC = area under curve, CI = confidence interval, OS = overall survival, RDW = red cell distribution width.

**FIGURE 3:**
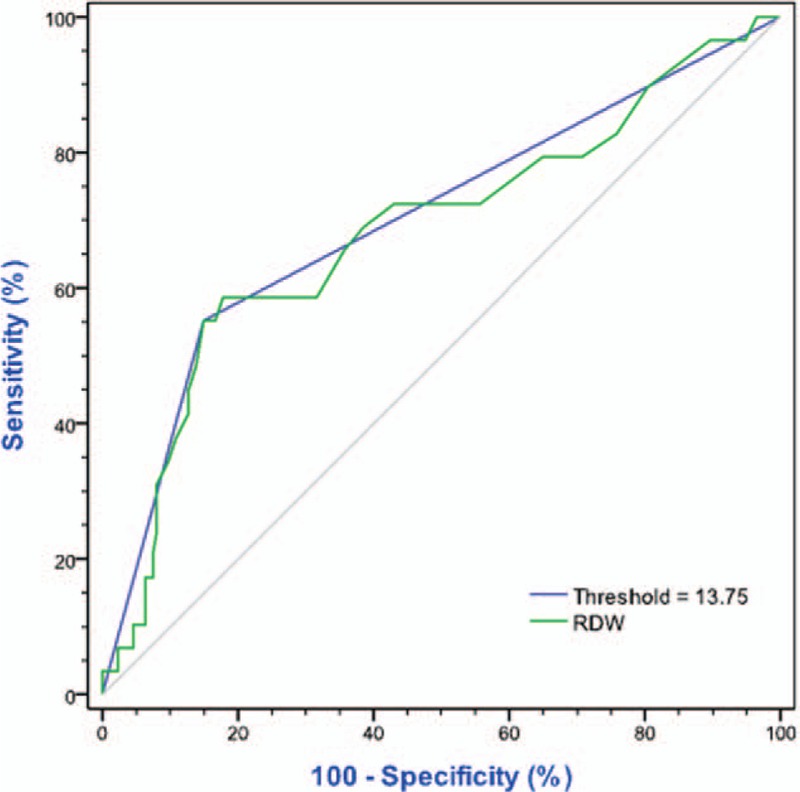
Receiver operating characteristics curve (ROC) analysis based on RDW for DFS. In this model, AUC was 0.685 (95% CI 0.596–0.800), *P* = 0.001. When threshold = 13.75%, the AUC = 0.701 (*P* = 0.001), and the sensitivity = 55.2% and specificity = 85.1%. AUC = area under curve, CI = confidence interval, DFS = disease-free survival, RDW = red cell distribution width.

The RDW was calculated from the blood routine test performed immediately after breast cancer diagnosis and before the initiation of any treatment (pretreatment RDW). The threshold of 13.75% was decided as the maximum (sensitivity + specificity) point according to largest the area under curve (AUC) of receiver operating characteristics curve (ROC; Figures 2 and 3). Patients were further divided into 2 groups: low RDW group (RDW ≤ 13.75%) and high RDW group (RDW > 13.75%).

### Statistical Analysis

OS was defined as the time from surgery to death. DFS was defined as the time from surgery to local-regional recurrences or distant metastases. Statistical analysis was performed using GraphPad Prism version 6.01 (GraphPad Software Inc., La Jolla, CA, USA) and IBM SPSS Statistic v21.0 (SPSS Inc., Chicago, IL, USA). The Kolmogorov–Smirnov tests were used to test for normality within ages and the values of RDW. They were abnormal distribution and were expressed as the median (range) and compared using the Wilcoxon rank-sum test. Categorical variables were compared using the Chi-squared test. The censoring time was defined as the last follow-up time. And Kaplan–Meier survival curves with log-rank tests and Cox proportional hazard regression analyses were used to compare the survival rates with clinical and pathologic factors. Variables with *P* < 0.05 in the univariate Cox regression analysis were progressed to a multivariate analysis using forward stepwise selection. *P* < 0.05 was considered statistically significant and all *P* values were 2-tailed.

## RESULTS

There were 203 young women enrolled with operative breast cancer for this retrospective study. The median age was 37 years old. And the median follow-up time was 48 months (range from 4 to 85 months).

**FIGURE 4:**
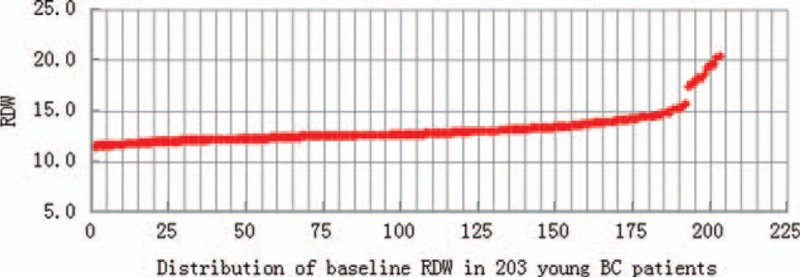
Distribution of the baseline RDW in the peripheral blood of 203 patients with breast cancer. RDW = red cell distribution width.

The distribution range of pretreatment RDW was shown in Figure 4 (range from 12% to 20%, median 12.70%). When the value of RDW cut into 2 groups (low RDW group and high RDW group) by 13.75%, the AUC became the largest, in ROC analyses based on RDW for OS and DFS. As shown in Figure 2, the sensitivity = 72.7% and specificity = 82.3% for OS, when cut-off value = 13.75% (*P* = 0.002). While shown in Figure 3, the sensitivity = 55.2% and specificity = 85.1% for DFS (*P* = 0.001).

There were 161 patients with low RDW (≤13.75%) and 42 patients with high RDW (>13.75%). And there were no significant correlations among RDW and lots of clinical pathological factors, including age, peritumoral vascular invasion (PVI), ER status, PR status, HER-2 status, Ki-67, different types of surgery, whether taking chemotherapy or not, and chemotherapy regimens (Table 1). However, in high RDW group, there seemed to be more patients with larger tumor size (55.3% vs 44.7%, 28.6% vs 71.4%, respectively, *P* = 0.002), positive lymph node metastases (57.8% vs 42.2%, 35.7% vs 64.3%, respectively, *P* = 0.011), and advanced stages (*P* = 0.004).

**TABLE 1:**
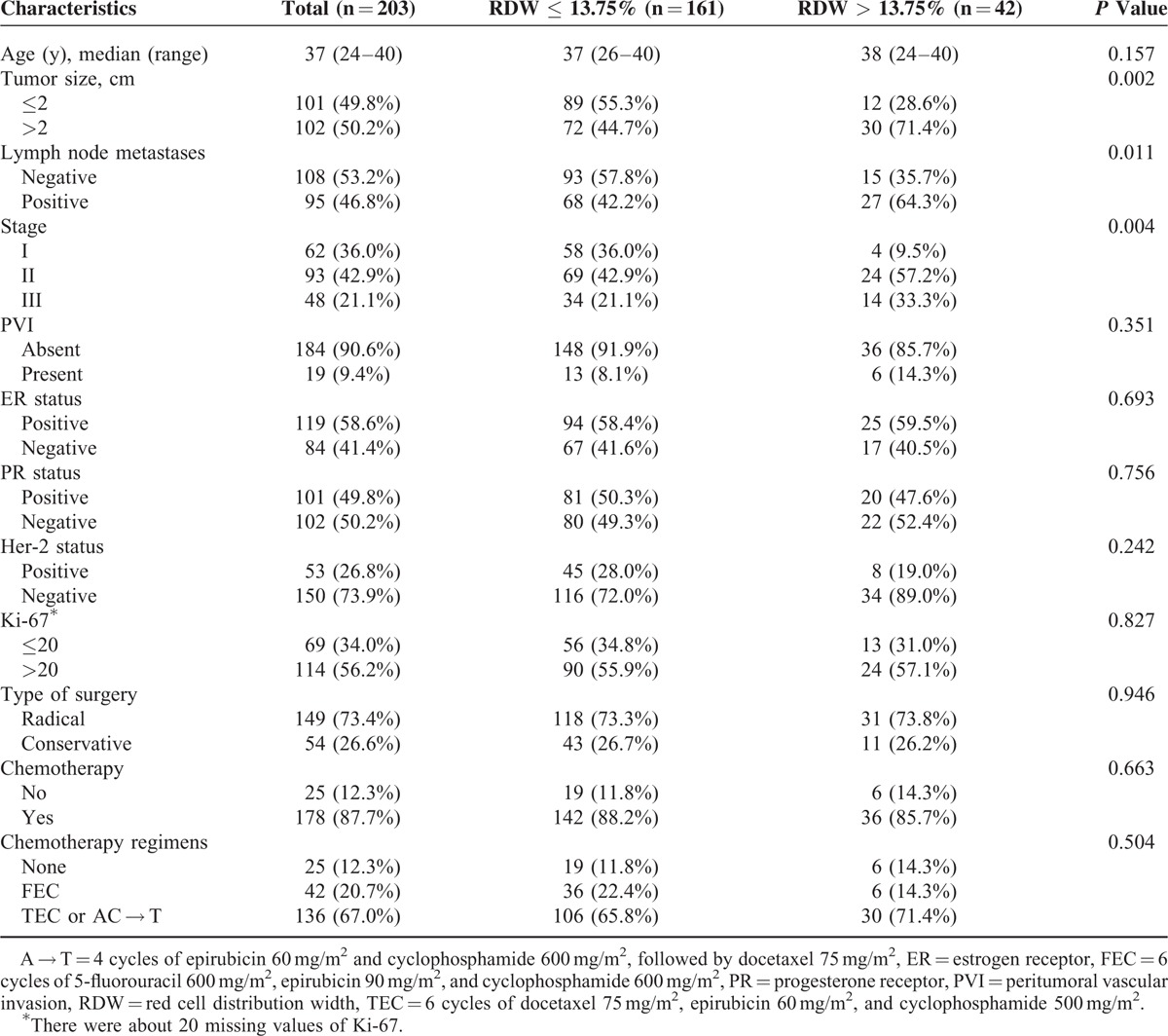
Clinicopathological Characteristics of Young Patients With Breast Cancer

**FIGURE 5:**
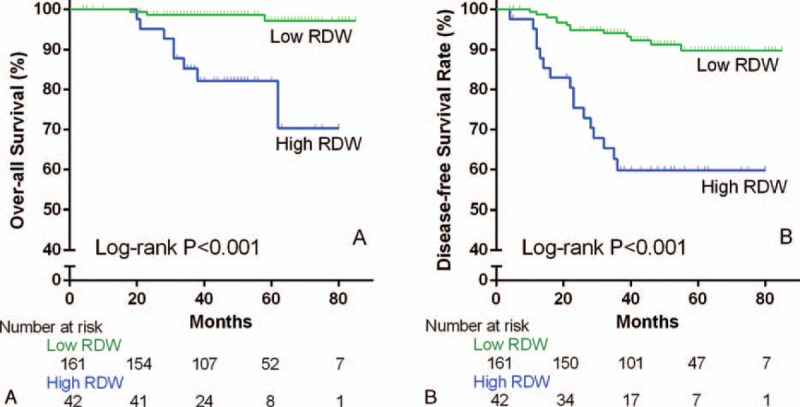
(A) OS of young women with breast cancer based on DRW (*P* < 0.001). (B) DFS of young women with breast cancer based on DRW (*P* < 0.001). DFS = disease-free survival, OS = overall survival, RDW = red cell distribution width.

As shown in Figure 5A, patients with high RDW appeared significantly lower OS rate than those with low RDW (5-year OS rate, 70.34% vs 97.14%, *P* < 0.001). And as shown in Figure 5B, high RDW group revealed lower DFS rate either (5-year DFS rate, 58.44% vs 91.78%, *P* < 0.001).

The Cox regression univariable analysis for young women revealed that high pretreatment RDW (>13.75%) and PVI presentation were associated with poor OS, with hazard ratio (HR) 11.674 (95% confidence interval [CI] 3.068–44.413, *P* < 0.001) and 6.777 (95% CI 1.981–23.177, *P* = 0.002), respectively. And positive ER and PR status were protectively predictive factors of OS, with HR 0.257 (95% CI 0.068–0.969) and 0.100 (95% CI 0.013–0.782), respectively. After multivariate statistical analysis, high RDW, PVI presentation, and positive PR status were independently prognostic factors for OS (all *P* value < 0.05; Table 2).

**TABLE 2:**
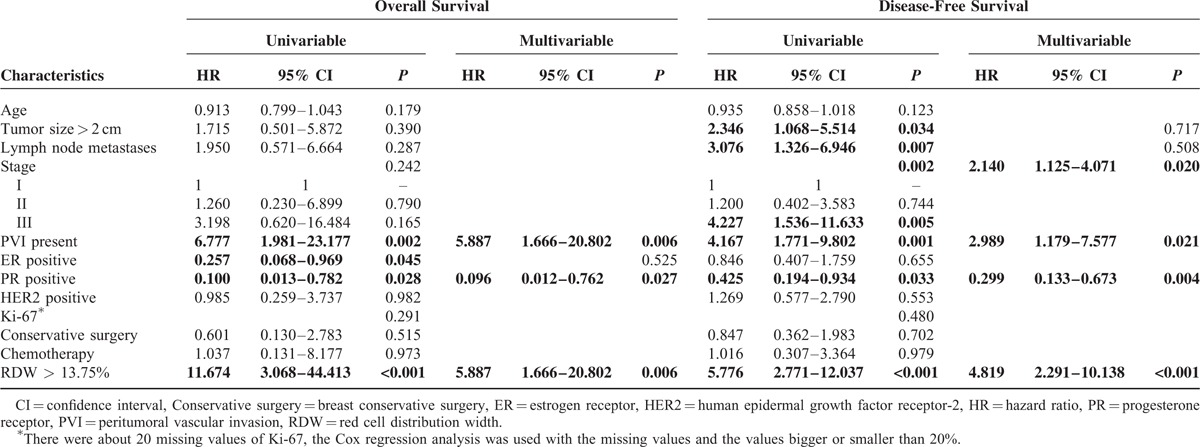
Cox Regression Analyses of Survival for Young Patients With Breast Cancer

The Cox regression univariable analysis also indicated that high pretreatment RDW, larger tumor size (> 2 cm), lymph node metastases presentation, more advanced stage, and PVI presentation were related with poor DFS (all HR > 1, *P* value < 0.05). And positive PR status was correlative with better DFS, with HR 0.425 (95% CI 0.194–0.934, *P* = 0.033). The multivariate analysis showed that high pretreatment RDW, positive PR status, more advanced stage, and PVI presentation were independently prognosis factors for DFS (all *P* value < 0.05; Table 2).

## DISCUSSION

Young women with breast cancer are more likely to present with more aggressive disease and have poorer outcome compared with their older counterparts.^7^ Inflammation in the tumor microenvironment promotes tumor growth, invasion, angiogenesis, and eventually metastasis.^8–10,22^ Elevated inflammatory markers, such as C-reactive protein (CRP), neutrophil to lymphocyte (N/L) ratio, interleukin-6, have been related to poorer survival among breast cancer patients.^23–26^ Furthermore, inflammation could bring changes in red blood cell maturation by disturbing the red cell membrane, leading to increased RDW.^27^ As a routinely available marker of the systemic inflammatory response, RDW has recently been shown to negatively influence the clinical outcome in various cancer entities.^16,17,20^

Our study demonstrated that an elevated pretreatment RDW was an independent factor of poor survival in young women with breast cancer. This result is in accordance with the previous report regarding breast cancer.^28^ Moreover, we found that 13.75% may be a suitable threshold for predicting recurrence or death with ROC test (*P* for OS = 0.002; *P* for DFS = 0.001, respectively). All the specificities were nearly 85%, suggesting that more attention should be paid to the patient with higher preoperational RDW. However, the sensitivity of recurrence prediction was too low to recommend the aggressive treatment directly. Combined with other predictive indicators, such as preoperational BMI or N/L ratio, the prognostic prediction of RDW might be more significant in young patients with breast caner.^28,29^

Moreover, to our knowledge, the present study is the first to analyze RDW in young women with breast cancer, suggesting that increased pretreatment RDW may be associated with worse prognosis in young women with breast cancer. Also, taking into account that RDW is easily available in routine blood tests and its cost-effective advantage, the role of the RDW could represent a new accurate and reproducible laboratory index to identify patients with worse prognosis in young women with breast cancer. However, further prospective studies are needed to evaluate the potential role of RDW in guiding treatment decisions.

In addition, our data are consistent with the study by Seretis et al,^19^ in which RDW has been reported to be a useful biomarker to distinguish between benign or malignant breast tumors. Moreover, RDW elevation is significantly correlated with larger primary tumors, higher number of infiltrated axillary lymph nodes, and advanced stages. The possible explanation could be that more aggressive tumors may trigger an extended inflammatory reaction during their progression, with increased levels of circulating cytokines, such as interleukin-6, CRP, and N/L ratio.^23–26^ These suggested that RDW may be a potential biomarker of cancer growth and metastatic activity in breast cancer. However, we did not identify any relationship between RDW and HER-2 overexpression. These differences might be attributed to the different sample of the patients enrolled in our study.

There are some limitations in our study. It was conducted in a single center, and it is a retrospective analysis on a small number of patients. Thus, further multicenter prospective studies which contain more patients are needed.

In conclusion, our present study revealed that pretreatment DRW may be associated with DFS and OS in young women with breast cancer. Given that DRW is readily available biomarkers in clinical settings, further validation and feasibility studies are warranted to determine the added value of DRW in the prognostication of breast cancer occurs in young women.
